# Weekend admissions and outcomes in patients with pneumonia: a systematic review and meta-analysis

**DOI:** 10.3389/fpubh.2023.1248952

**Published:** 2024-01-17

**Authors:** Jiayao Lu, Jing Yang, Xiaofei Cai

**Affiliations:** Department of 12 Ward, Huzhou Third Municipal Hospital, The Affiliated Hospital of Huzhou University, Zhejiang, Huzhou, China

**Keywords:** pneumonia, weekend effect, mortality, readmission, intensive care, systematic review, meta-analysis

## Abstract

**Background:**

To document pooled evidence on the association between weekend hospital admissions and the potential risks of mortality, intensive care requirements, and readmission among patients with pneumonia.

**Methods:**

We performed a systematic search across the PubMed, EMBASE, and Scopus databases. We collected observational studies exploring the association between weekend admissions and outcomes of interest in patients with pneumonia. To analyze the data, we used a random effects model and expressed the effect sizes as pooled odds ratios (ORs) accompanied by their respective 95% confidence intervals (CIs).

**Results:**

The analysis comprised data from 13 retrospective studies. Compared to patients admitted on weekdays, those admitted during the weekend had a non-statistically significant marginally higher risk of in-hospital mortality (OR, 1.02; 95% CI, 1.00, 1.04) but similar 30-day mortality after admission (OR, 1.03; 95% CI, 0.97, 1.10), and similar risks of admission to intensive care unit (OR, 1.04; 95% CI, 0.98, 1.11) and re-admission (OR, 0.85; 95% CI, 0.65–1.12).

**Conclusion:**

Our findings do not support the presence of a “weekend effect” in patients with pneumonia.

**Systematic review registration:**

PROSPERO, identifier CRD42023425802, https://www.crd.york.ac.uk/prospero/.

## Introduction

Pneumonia is a significant global health concern with substantial morbidity and mortality worldwide (1–3). Pneumonia is a leading cause of illness and death, particularly among vulnerable populations such as young children, older adult, and individuals with underlying health conditions (4, 5). Pneumonia is associated with significant healthcare use, including hospitalizations, intensive care unit (ICU) stays, and the need for respiratory support and antibiotics (6–8).

The idea of the “weekend effect” refers to the increased risk of mortality in patients admitted to hospitals on Saturdays or Sundays compared to the risks in patients admitted on weekdays (9, 10). Researchers, policymakers, and healthcare professionals have tried to understand the underlying mechanisms of the weekend effect, and several factors have been suggested as potential contributors, including differences in staffing levels, reduced availability of certain services or resources, delayed diagnostic tests, and limited access to specialized interventions during weekends (11–13). Studies have shed light on this intriguing pattern (9, 10, 14, 15). Zhou et al. performed a comprehensive meta-analysis of 140 studies to examine the association between admission during off-hours or on weekends and the risk of mortality across various diseases (16). Their findings revealed a substantial correlation between off-hour admissions and elevated mortality rates for specific conditions (aortic aneurysm, breast cancer, pulmonary embolism, arrhythmia, and cardiac arrest). No significant association was observed for other conditions such as hip fracture, pneumonia, intestinal obstruction, or trauma. However, when considering ~28 diseases together, off-hour admission demonstrated a consistent association with increased mortality (16). This above-mentioned review was published in 2016 and included seven unique studies on pneumonia. Since then, other studies looking at the effect of timing of admission on the survival of patients with pneumonia have been published. Thus, we decided to update the evidence with findings of newer studies. Our meta-analysis aims to pool findings from all available studies to present updated evidence on the impact of weekend admission on mortality, risk of admission to intensive care unit (ICU), and readmission in patients with pneumonia.

## Methods

### Process for selection of studies

We conducted a comprehensive search of three electronic databases (PubMed, Embase, and Scopus) to identify relevant studies for our analysis. The search encompassed articles published from the inception of these databases up to April 30, 2023. We designed the search strategy using a combination of medical subject headings (MeSH) and free-text terms, focusing on topics such as pneumonia, off-hours admission, weekend admission, and outcomes. The search strategy included the following entries: (off-hours admission OR, weekend admission OR, weekday admission) AND (pneumonia OR, respiratory infection OR, lung infection OR, pneum^*^) AND (clinical outcome OR, mortality OR, death OR, survival OR, readmission OR, intensive care). Moreover, we conducted a thorough screening of the reference lists of the relevant articles and systematic reviews to identify any additional studies that met our predefined inclusion criteria. This process allowed us to capture any potentially relevant studies. The need for ethical approval was waived due to the nature of this systematic review and meta-analysis based on existing literature. To ensure the transparency and completeness of our methodology and findings, we followed the guidelines set forth by PRISMA (17). This protocol was registered at PROSPERO, No. CRD42023425802.

To ensure the quality and reliability of our study, we implemented a robust process that involved two independent reviewers meticulously screening all identified studies for inclusion. This screening was carried out on the basis of predetermined eligibility criteria that were established prior to initiating the study. Our primary focus was on observational studies that specifically examined the association between weekend admission (as opposed to weekday admission) and various outcomes of interest in individuals admitted for pneumonia within healthcare facilities. These outcomes primarily encompassed mortality rates, the risk of admission to the intensive care unit (ICU), and the risk of readmission. To ensure consistency and adherence to our selection criteria, we identified studies reporting on at least one of the specified outcomes. Moreover, we limited our inclusion to studies published in English. During the literature search, we applied specific exclusion criteria to ensure the relevance and appropriateness of the selected studies. Any studies containing duplicate or overlapping data were excluded from consideration. In addition, we also excluded case reports, editorials, letters, and conference abstracts.

In case of disagreements between the two reviewers during the screening process, discussions were held to reach a consensus. If necessary, a third expert reviewer was consulted. Finally, after the initial screening, we retrieved and carefully assessed the full texts of the potentially eligible studies to ensure that only the most relevant and appropriate studies were incorporated into our analysis.

### Extraction of relevant data from included studies

Two authors extracted the data independently using a standardized form to ensure consistency and uniformity. Any discrepancies during the data extraction phase were resolved through discussion between the two authors to reach a consensus. To assess the risk of bias within the included studies, we used the Newcastle-Ottawa Scale (NOS) (18), a widely accepted tool for evaluating the quality and potential bias of the selected studies. Again, disagreements were resolved during discussions or after consulting with a third expert. This collaborative approach ensured a comprehensive and reliable evaluation of the included studies, minimizing the potential for bias and enhancing the overall quality of our study.

### Data analysis

To estimate the pooled effect sizes (odd's ratios, ORs) and their corresponding 95% confidence intervals (CIs) for each outcome of interest, we applied a random-effects model. This statistical approach considers both within-study and between-study variability (19). We calculated the overall effect estimate by first obtaining a weighted average of the effect sizes from each individual study. The weights were determined on the basis of the inverse of the variance of each effect size. This weighting scheme gives more emphasis to studies with smaller variances, and provides a precise estimate of the overall effect (19).

To assess the presence of statistical heterogeneity, we calculated the *I*^2^ statistic, which quantifies the proportion of total variability across studies that can be attributed to heterogeneity rather than chance (20). Moreover, we conducted an evaluation of publication bias using Egger's test and visual inspection of funnel plots (21). We considered *P*-values lower than 0.05 as indicative of statistically significant publication bias.

## Results

**Figure 1:**
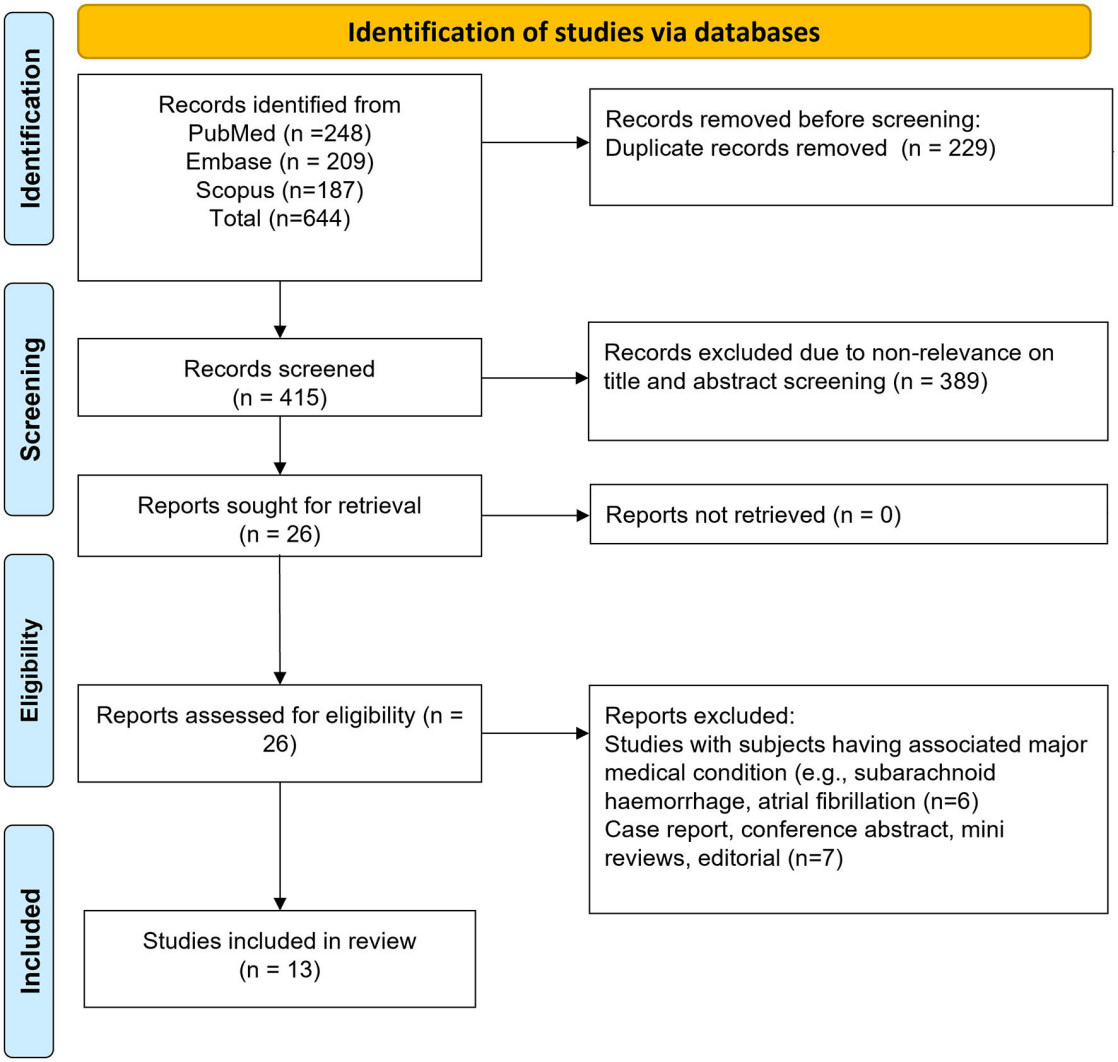
Selection process of studies included in the review.

We initially identified 644 studies through our search strategy. After removing 229 duplicates, we were left with 415 unique studies. We further screened the titles and abstracts of these studies and excluded 389 of them. We proceeded to the detailed review of the full texts of the remaining 26 studies, leading to the exclusion of an additional 13 studies. Ultimately, our meta-analysis included 13 studies, as illustrated in Figure 1 (22–34).

The 13 retrospective studies included comprised data from 14,56,735 patients (Table 1). Three studies were conducted in United Kingdom (UK), two in Australia and Canada, and one each in Kenya, USA, Japan, Taiwan, Denmark, and Portugal (Table 1). All studies, except one, included only adults. The quality assessment score varied between 6 and 8, out of the maximum attainable score of 9. The mean NOS score of the included studies was 7.1 (Table 1).

**Table 1:** Included studies and their key characteristics.

**References**	**Study design**	**Country**	**Patient characteristics**	**Sample size**	**Outcomes (weekends, compared to weekdays)**	**Newcastle Ottawa quality score**
Bell and Redelmeier (22)	R	Canada	Women (51%); most older than 20 years (80%)	98,318	Mortality (in-hospital): OR, 1.03 (95% CI, 0.98, 1.08)	8
Cram et al. (23)	R	USA	Mean age, 66 years; women (~52%); white race (75%)	14,199	Mortality (in-hospital): OR, 1.00 (95% CI, 0.91, 1.10)	7
Schmulewitz et al. (24)	R	UK	Mean age of 65 years; women (45%)	561	Mortality (in-hospital): OR, 0.99 (95% CI, 0.27, 3.01) Readmission: OR, 0.78 (95% CI, 0.50, 1.20)	6
Aylin et al. (25)	R	UK	Age group 15–64 years (~50%); women (53%)	102,465	Mortality (in-hospital): OR, 1.00 (95% CI, 0.97, 1.04)	7
Chang and Tung (26)	R	Taiwan	Most older than 75 years (>50%) and men (>60%); ~16% had at least 1 previous ICU admission	788,011	Mortality (30-day): OR, 1.03 (95% CI, 1.01, 1.05)	6
Gathara et al. (27)	R	Kenya	Median (IQR) age, 8 months (5–13) months; most between 2 and 11 months (69%); men (55%); mild disease (60%)	1,825	Mortality (in-hospital): OR, 1.15 (95% CI, 0.90, 1.45)	7
Suissa et al. (28)	R	Canada	Mean age, 75 years; men (55%); 28% with prior hospitalization	323,895	Mortality (in-hospital): OR, 1.04 (95% CI, 1.00, 1.08)	7
Vest-Hansen et al. (29)	R	Denmark	Median age, 75 years; women (~51%); associated comorbidities in one-fifth of patients	11,858	Mortality (30-day): OR, 1.22 (95% CI, 1.05, 1.43)	8
Uematsu et al. (30)	R	Japan	Median age, 83 years; women (38%); with severe pneumonia (57%); associated comorbidities in one third of patients	23,532	Mortality (in-hospital): OR, 1.10 (95% CI, 1.02, 1.19)	6
Cortes et al. (31)	R	Portugal	Median age, 84 years; men (55%)	53,876	Mortality (in-hospital): OR, 1.02 (95% CI, 0.97, 1.06) Admission to ICU: OR, 1.04 (95% CI, 0.96, 1.11)	7
Baldwin et al. (32)	R	Australia	Median age, 75 years; most older than 60 years (~80%); men (55%)	44,508	Mortality (30-day): OR, 1.06 (95% CI, 0.98, 1.14)	8
Lawrence et al. (33)	R	UK	Mean age, 72 years; men (49%); associated comorbidities- chronic heart disease (23%), chronic kidney disease (8%), chronic obstructive pulmonary disease (24%)	32,984	Mortality (in-hospital): OR, 0.96 (95% CI, 0.87, 1.06) Mortality (30-day): OR, 0.94 (95% CI, 0.88, 1.01) Admission to ICU: OR, 1.05 (95% CI, 0.95, 1.16) Readmission: OR, 0.99 (95% CI, 0.92, 1.07)	8
Milevski et al. (34)	R	Australia	Most older than 70 years (78%); men (60%)	753	Mortality (30-day): OR, 1.16 (95% CI, 0.71, 1.89) Readmission: OR, 0.64 (95% CI, 0.40, 1.01)	7

R, Retrospective.

Table 2 provides additional insights into the included studies, particularly concerning the type of pneumonia, administered treatments, details about health facilities, and variables adjusted in the analytic model. Among the 13 studies, 9 simply specified “pneumonia” without differentiating between bacterial or viral origins. One study specifically identified cases as “acute bacterial pneumonia” (31), while the remaining three studies focused on “community-acquired pneumonia” (30, 33, 34). Regarding the treatment or quality of care given to pneumonia patients and whether there were distinctions between weekend and weekday admissions, a majority of studies did not furnish this information. One study reported no significant disparities in the quality of care indicators between weekends and weekdays (27). In contrast, another study noted that the weekend admission group exhibited significantly lower rates of microbiological testing, such as sputum culture and urine antigen tests, compared to the weekday admission group (30). This same study also revealed that X-ray tests and blood tests (urine nitrogen, C-reactive protein, and complete blood count) were marginally more frequent in the weekend admission group (30). Yet another study found no significant differences between weekends and weekdays in terms of quality of care indicators, except for a lower proportion of weekend-admitted patients receiving reviews by senior medical personnel within 12 h of admission (33). In terms of the study settings, six investigations were conducted in acute care hospitals, while four were carried out in large tertiary care or academic teaching hospitals.

**Table 2:** Additional details on the included studies.

**References**	**Type of pneumonia**	**Patient characteristics**	**Treatment provided**	**Quality of hospital/staff/equipment**	**Adjustment**
Bell and Redelmeier (22)	Pneumonia, organism not specified	Similar sex distribution: Charlson score for comorbidity similar across both groups i.e., those admitted on weekdays and weekend	Not provided; the data were used from the Canadian Institution for Health Information, a large database for a large number of disease conditions and specific details on treatment were not provided in the study	Patients admitted to acute care hospitals through emergency department; proportion admitted to teaching hospitals similar in both groups	For age, sex, and the score on the Charlson comorbidity index
Cram et al. (23)	Pneumonia, organism not specified	Similar sex distribution: similar proportion in terms of race distribution: Charlson score for comorbidity similar across both groups i.e., those admitted on weekdays and weekend	Not provided; data from California Office of Statewide Health Planning and Development Discharge Data File were used and specific details on treatment were not provided in the study	Acute care hospitals (excluding Veterans Administration hospitals)	For demographic characteristics (age, sex, and race) and comorbidity
Schmulewitz et al. (24)	Pneumonia, organism not specified	Higher proportion of females in the weekend group	Not provided; hospital admission data for the Royal Infirmary of Edinburgh via the Lothian NHS Trust (PAS) Database were used	Medical assessment units (MAU) were appropriately resourced to provide round the clock services throughout the year with adequate staff numbers, including access to diagnostics and the allied health specialties. The MAU had the same consultants and junior staffing ratios at weekends and weekdays. Access to diagnostic radiology for investigations such as computed tomography of head and chest and ultrasound were similar throughout the 7-day period.	Adjusted for age and sex
Aylin et al. (25)	Pneumonia, organism not specified	Statistically significant differences in age (older subjects in weekend group), sex (more males in weekend group), and the Charlson comorbidity index (lower in weekend group); these differences were, however, small	Not provided; data from all acute public hospitals in England from the NHS Wide Clearing Service was used	All acute public hospitals in England	For age, sex, and comorbidity
Chang and Tung (26)	Pneumonia, organism not specified	Majority were older than 75 years (>50%) and men (>60%); around 15% required admission to intensive care unit; around 10% required mechanical ventilation	Not provided; nationwide longitudinal population- based data was used and specific details of management not provided	General acute care hospitals throughout Taiwan; unaccredited hospitals were excluded; mean physician volume increased from 62 cases in 1997 to 106 cases in 2008; >70% cases were managed by internal medicine department	For gender, age, illness severity and comorbidities and health care factors such as physician characteristics (age and specialty), hospital characteristics (teaching status, geographic location) and time trend.
Gathara et al. (27)	Pneumonia, organism not specified	Similar age, sex and comorbidities distribution	No significant differences between weekends and weekdays for the quality of care indicators (i.e., adequate assessment, appropriate antibiotic and dosage consistent with guidelines)	Large tertiary care hospital; hospital had four general pediatric wards each with 60 beds and bed occupancy was often over 100%. Most of clinical in-patient care was provided by 60–75 trainee pediatricians and are normally supervised by 25 pediatricians. There were 126 qualified nurses on the general pediatric wards. At the time of the study, each ward had 5–8 pediatricians, 5–8 pediatric trainees per day and 5–6 nurses per shift and this staff distribution did not differ on weekends and weekdays.	For age, gender, comorbidities and disease severity
Suissa et al. (28)	Pneumonia, organism not specified	Mean age, 75 years; men (55%)	Data from computerized databases of the Régie de l'Assurance Maladie du Québec (RAMQ), Quebec, Canada were used, and specific treatment offered to the patients were not provided in the study	Patients admitted to acute care hospitals	For age, sex, calendar year of admission, admission day, prior hospitalization and comorbidities
Vest-Hansen et al. (29)	Pneumonia, organism not specified	Similar age, sex and comorbidities distribution; higher proportion of admissions from outpatient department in weekday group and from emergency department in weekend group	Not provided; data from Danish based registry used (Danish National Registry of Patients (DNRP).); study did not provide information on treatment	All acute hospital admissions to medical departments in Denmark; specifics on infrastructure, quality of care and staff available is not mentioned in the study	For age, sex, and comorbidity
Uematsu et al. (30)	Severe community acquired pneumonia	Similar age, gender distribution, severity of pneumonia and comorbidities across the two groups	Diagnosis Procedure Combination (DPC) database was used; weekend admission group had significantly lower rates of microbiological testing (such as sputum culture and urine antigen tests) when compared with the weekday admission group. However, Xray tests and blood tests (urine nitrogen, C-reactive protein and complete blood count) were performed slightly more often in the weekend admission group.	Admission to teaching hospital (70%); median hospital volume (pneumonia cases/year) of 33	For patient age, gender, severity of pneumonia, comorbidities, ambulance use, non-elective admission and referral from other facilities
Cortes et al. (31)	Acute bacterial pneumonia	Median age of patients admitted during the weekend was slightly higher than that of patients admitted during the weekdays; similar gender distribution	In-patient database from a large tertiary center in Portugal was used; specific details of management not provided; overall around 6% had admission to intensive care unit (ICU) and similar proportion of subjects in both groups had admission to ICU	Admission to large tertiary care hospital.	For age, gender and ICU treatment
Baldwin et al. (32)	Pneumonia, organism not specified	Similar age, sex and comorbidities distribution; higher proportion admitted through emergency department in weekend group	All admissions to New South Wales (NSW) public and private hospitals were considered; no information provided on treatment provided	Public and private hospitals; no additional details on infrastructure, availability of services and staff provided	For age, sex, year and comorbidities
Lawrence et al. (33)	Community acquired pneumonia	Slightly older age and higher proportion with severe disease and admission through emergency department in weekend group; similar sex distribution; similar comorbidities distribution except for high proportion with cerebrovascular disease in weekend group	No significant differences between weekends and weekdays for the quality of care indicators except for lower proportion of patients admitted on weekend receiving review by senior medical personnel within 12 hours of admission.	Aggregate data from six British Thoracic Society national adult CAP audits; no additional details on infrastructure, availability of services and staff provided	For age, presence or absence of comorbidities and admitting hospital
Milevski et al. (34)	Community acquired pneumonia	Individuals admitted on weekend likely to be older and required assistance to Mobilize; higher proportion were females, with comorbidities, ever smoked, higher number of previous hospital admissions and with lack of spousal support in the weekend group	No specific information provided on the compliance with pneumonia treatment guidelines	Two tertiary academic hospitals; patients admitted under a General Internal Medicine (GIM) unit; each of the two hospital campuses had four distinct GIM units, each of which was staffed by separate teams consisting of interns, registrars and a senior consultant physician; staffing was significantly reduced across allied health and medical disciplines on weekends. On weekends, there was one medical consultant physician that covered all units, assisted by ~25% of the junior medical staff that would be present on a weekday. Allied health services were significantly reduced on weekends as well as access to specialty nursing services	For age, sex, smoking status, residential status, language spoken at home, whether living with a partner or not, aggregate comorbidity burden, illness severity, pre-morbid mobility status, and number of admissions to the health service in the preceding 6-months

**Figure 2:**
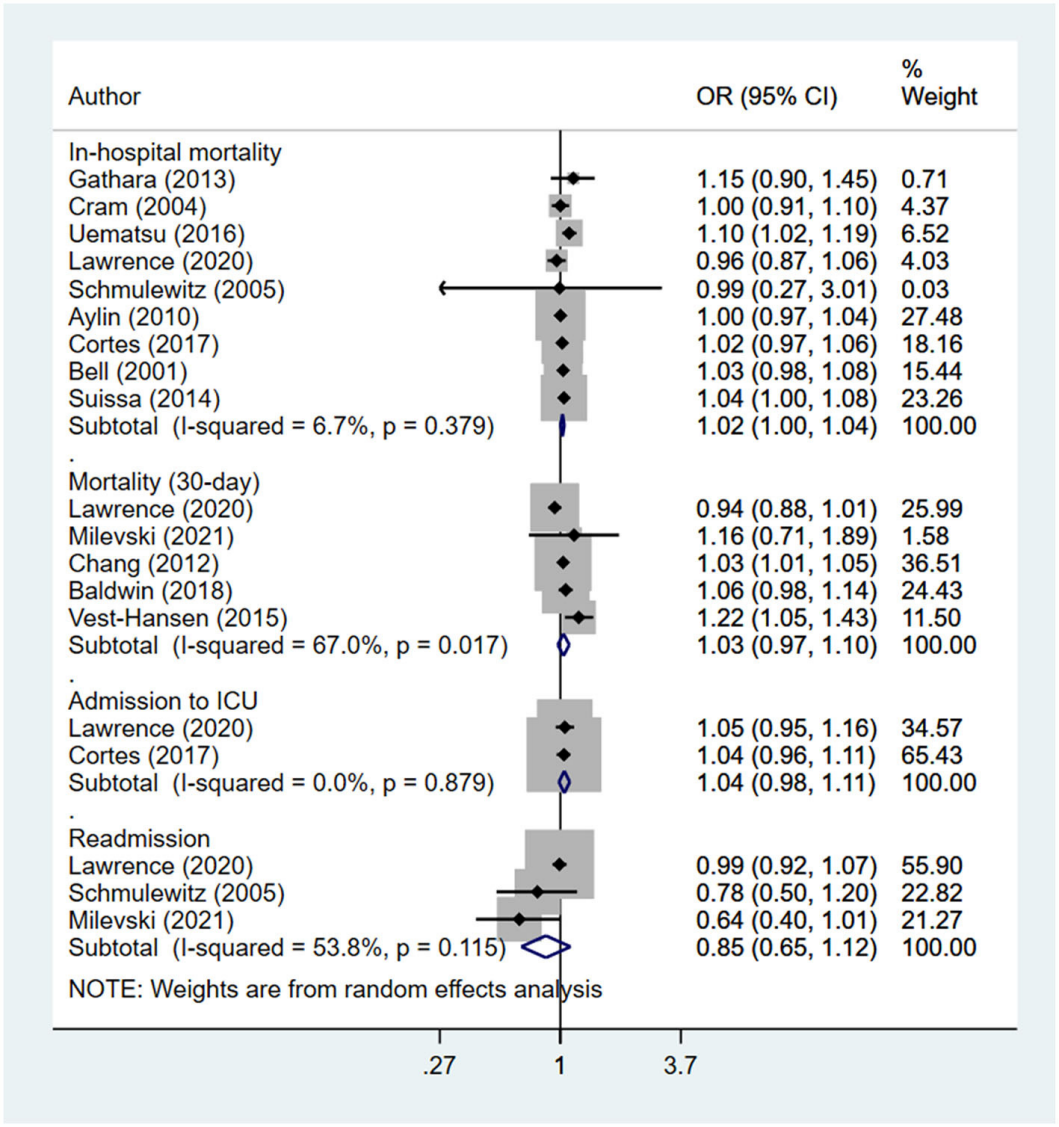
Comparison of outcomes between patients with pneumonia admitted during weekend and those admitted during weekdays.

Compared to patients admitted on a weekday, those with weekend admissions had a slightly higher risk of in-hospital mortality (OR, 1.02; 95% CI, 1.00, 1.04; *n* = 9; *I*^2^ = 6.7%), but similar risks of 30-day mortality (OR, 1.03; 95% CI, 0.97, 1.10; *n* = 5; *I*^2^ = 67%), admission to intensive care unit (OR, 1.04; 95% CI, 0.98, 1.11; *n* = 2; *I*^2^ = 0.0%), and being re-admitted (OR, 0.85; 95% CI, 0.65, 1.12; *n* = 3; *I*^2^ = 53.8%) (Figure 2). We found statistical evidence of publication bias for in-hospital mortality (*P* = 0.033), but not for other outcomes. The funnel plots for visual assessment of publication bias can be found in the Supplementary Figures 1–4.

## Discussion

In the current meta-analysis, we found that weekend admission for pneumonia was associated with a marginal increase in risk of in-hospital mortality, but not with risks of 30-day mortality, admission to intensive care unit, or being re-admitted. These findings confirm those reported by Zhou et al., who found no significant associations between off-hours admissions and the mortality risk in patients with pneumonia (16).

The weekend effect concept, which suggests that patients admitted during weekends experience poorer outcomes, may exhibit significant heterogeneity. The impact of the weekend effect probably depends on clinical conditions, hospital contexts, regional policies, and other factors that can differ considerably across geographical settings (11, 35–37). Studies have identified factors, including issues related to medical service accessibility and quality, limited access to specialized care, and reduced availability of certain procedures, which contribute to poor outcomes for patients admitted during off-hours or weekends (35–39). However, we found no elevated risk of mortality among patients with pneumonia admitted on weekends.

It is plausible that the weekend/off-hour effect is more pronounced for medical conditions that need additional specialized resources beyond standard care levels. The specialized healthcare resources level for the management of pneumonia is relatively low compared to the level needed for more critical conditions such as aortic aneurysms, pulmonary embolisms, arrhythmias, or cardiac arrests (40–42). Pneumonia can usually be readily diagnosed, and its treatment often follows standardized guidelines (40, 41). Therefore, pneumonia can be effectively managed by emergency medical staff without the immediate involvement of specialists. Thus, the impact of the weekend effect on patients with pneumonia may be less pronounced than the impact on conditions requiring a higher level of specialized care.

Most studies in our meta-analysis included vast amounts of routinely collected data as part of the patient care. By leveraging the information available on administrative data, researchers can gain deep insights into the weekend effect. However, improving coding standards and incorporating more refined parameters would help account for factors such as baseline disease severity, patient characteristics, and hospital workload (43, 44). The extensive datasets encompass various pre-defined fields, including demographic information, diagnoses, co-morbidities, and robust data on mortality and clinical outcomes (43, 44). While these data provide a valuable starting point, they offer limited specific information. Given the multifaceted nature of the weekend effect, researchers estimating mortality rates need to consider a multitude of factors, including the availability and quality of staff, as well as patient-level data. Research efforts need to shift focus from debating the mere existence of the weekend effect to delving deeper into its underlying causes and consequences to be able to implement innovative solutions and improve patient outcomes.

We are aware of certain limitations of the studies included. First, all of them were observational in nature, which makes them susceptible to potential confounding and bias. These studies cannot be used to establish a causal association between weekend admissions and outcomes in patients with pneumonia. Another potential limitation is the exclusion of unpublished studies and those published in languages other than English. With this decision, we may have introduced publication and language biases because we may have missed studies with negative or inconclusive results or those published in other languages. Incorporating a broader range of studies could provide a more comprehensive overview. Additionally, the underlying mechanisms that drive the association between weekend admissions and outcomes in patients with pneumonia remain unclear. The presence of high heterogeneity among some of the outcomes examined is worth noting, it could have arisen from differences in patient demographics, healthcare system characteristics, geographic locations, or other unaccounted factors. Further, performing statistical comparisons to analyze outcomes across various groups of hospitals, levels of staff arrangement, patients' characteristics, and types of pneumonia could have enhanced our understanding at a more detailed level. Unfortunately, not all studies furnished these essential details, and even among those that did, there was insufficient variation in relation to these factors. As a result, conducting statistical comparisons based on these variables was deemed unlikely to produce meaningful and actionable results. We acknowledge this limitation in our study. We recognize the importance of undertaking such an analysis, and to facilitate this, we advocate for future studies to present clear and comprehensive data on these variables. Finally, given that the analysis spans the pre- and during COVID-19 infection era, it is crucial to acknowledge this temporal context. Some of the included studies were published since the year 2019 and could have incorporated data from COVID-19 patients, thereby, introducing a unique aspect to the analysis. The nature of pneumonia cases, treatment protocols, and hospital resources may have been substantially altered to accommodate the challenges posed by the pandemic. Factors such as changes in healthcare infrastructure, and variations in treatment guidelines could have influenced the outcomes being studied. Healthcare systems worldwide underwent significant adaptations to address the surge in COVID-19 cases, potentially impacting the standardization of care and the comparability of results across different time points. Therefore, it is imperative to recognize this limitation when comparing findings across diverse publications with varying timeframes.

## Conclusion

Our findings do not support the presence of the weekend effect for patients with pneumonia. This suggests that outcomes in patients with pneumonia are probably not significantly influenced by the timing of admission. Therefore, healthcare providers and policymakers can focus on developing strategies that optimize care delivery and enhance patient outcomes consistently, regardless of the day or time of admission.

## Data availability statement

Publicly available datasets were analyzed in this study. This data can be found here: The datasets generated during and/or analyzed during the current study are available from the corresponding author on reasonable request.

## Author contributions

JL, XC, and JY wrote the manuscript and made the figures and approved the final revisions of the manuscript submitted for publication. JL and JY conducted the literature search, study selection, and analysis. All authors contributed to the article and approved the submitted version.

## Supplementary material

Supplementary Figure 1 Forest plot for in-hospital mortality.

Supplementary Figure 2 Forest plot for mortality at 30-day follow up.

Supplementary Figure 3 Forest plot for admission to ICU.

Supplementary Figure 4 Forest plot for readmission as an outcome.
