# Supplementary figures and images for: Weekend admissions and outcomes in patients with pneumonia: a systematic review and meta-analysis
Source: Front Public Health. 2024 Jan 17;11:1248952. doi: 10.3389/fpubh.2023.1248952 (PMC10832039; doi:10.3389/fpubh.2023.1248952)

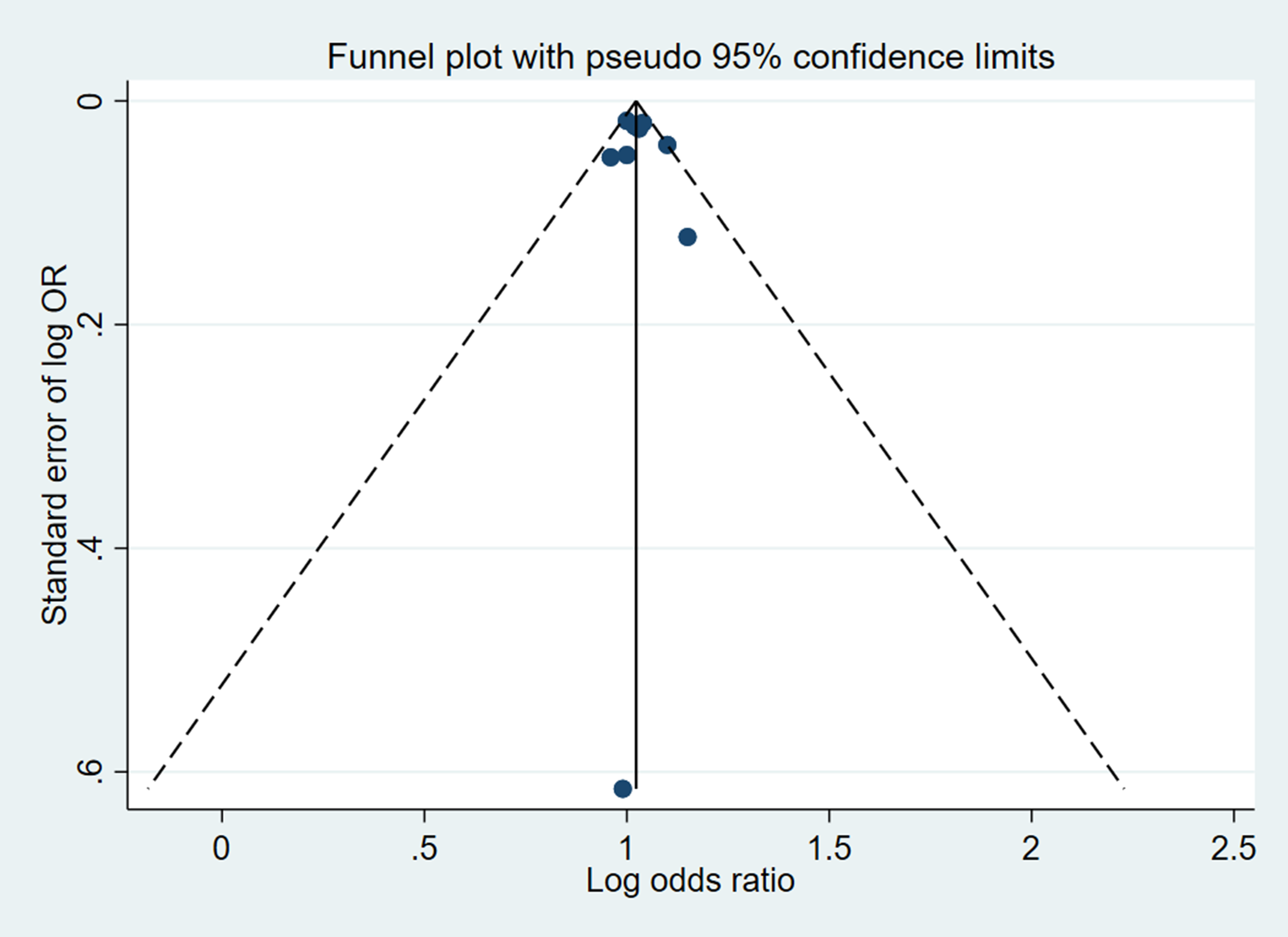

Supplement: Supplementary Figure 1 — Forest plot for in-hospital mortality. [file Image_1.TIF]

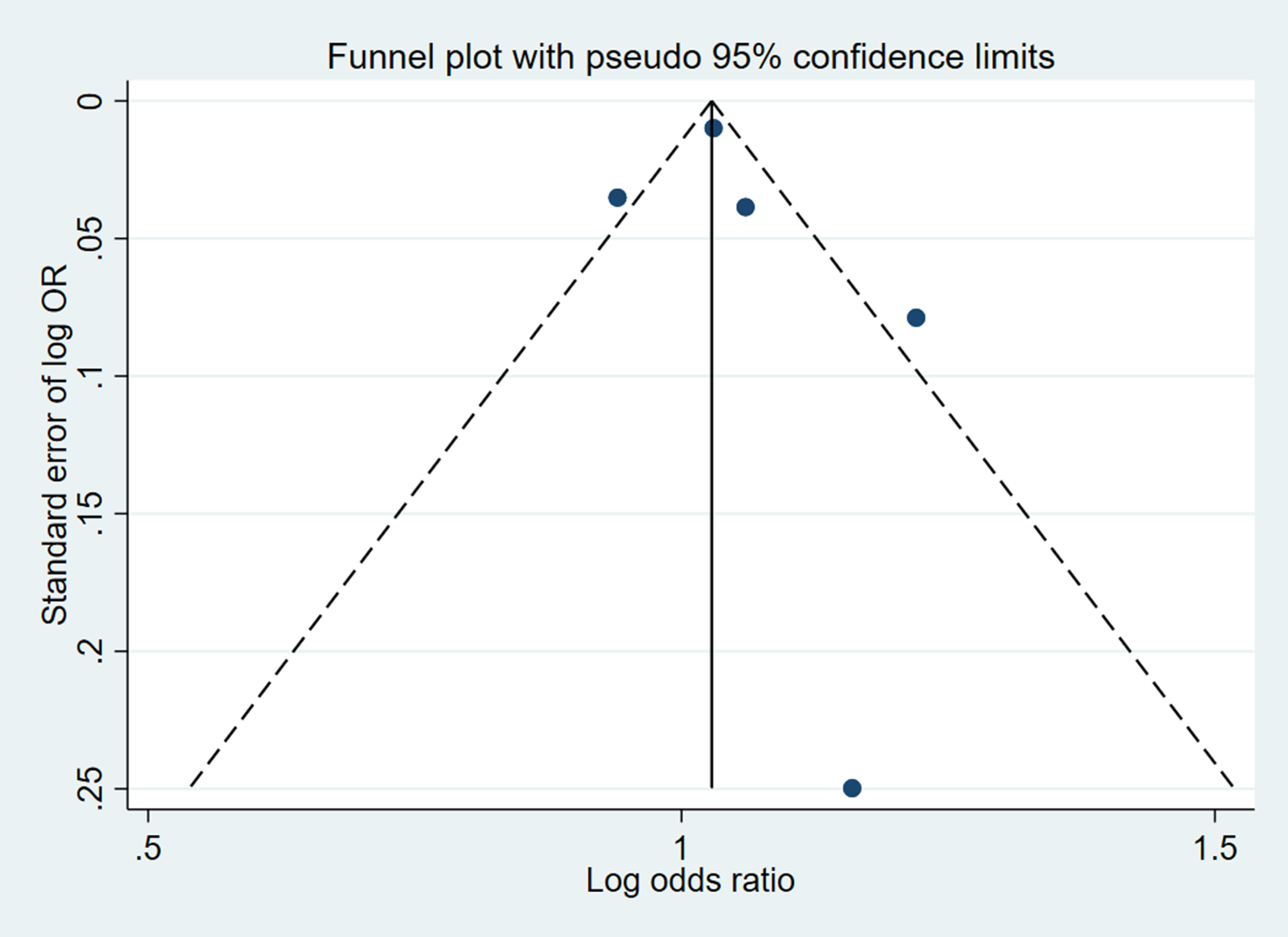

Supplement: Supplementary Figure 2 — Forest plot for mortality at 30-day follow up. [file Image_2.TIF]

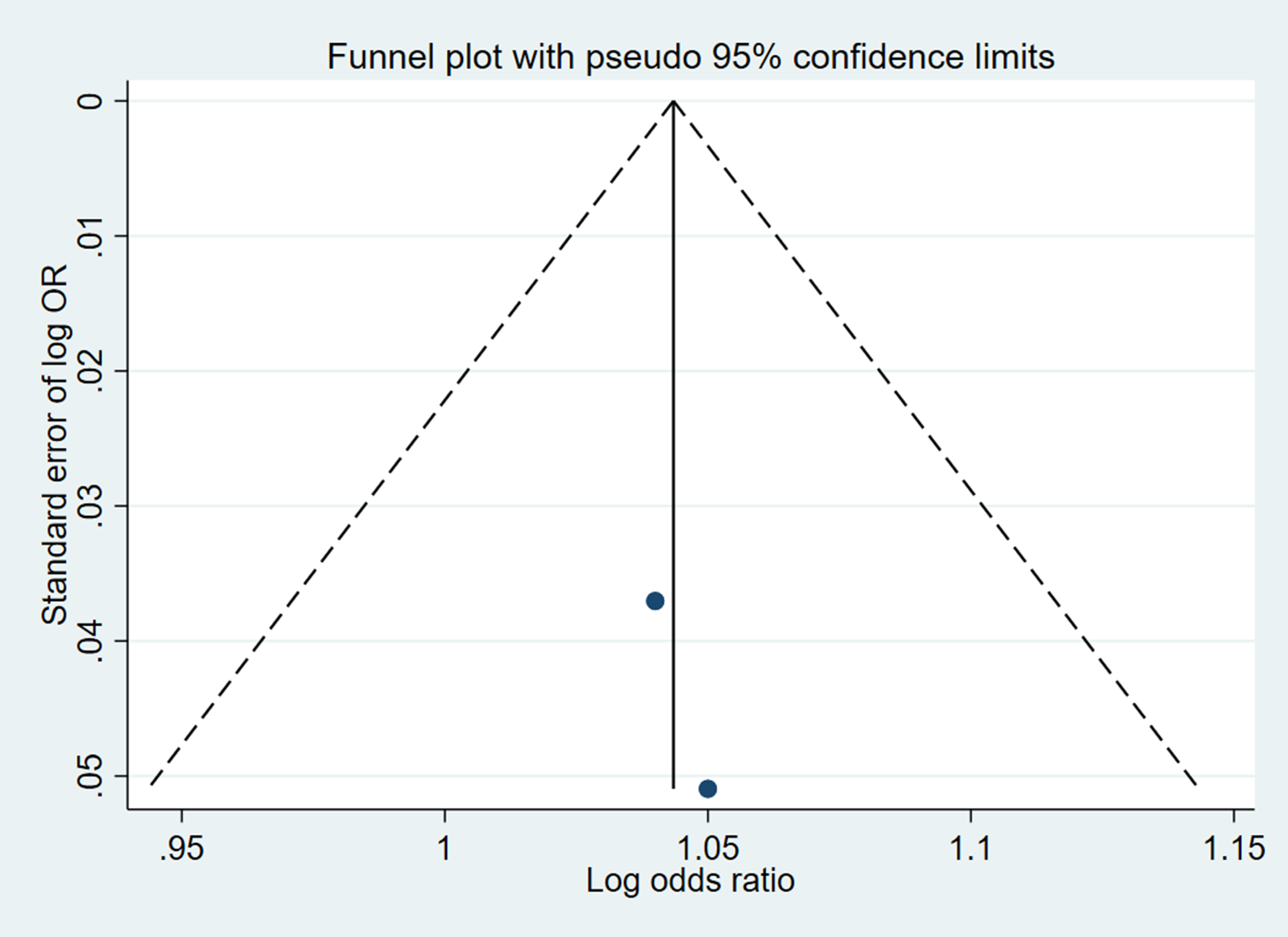

Supplement: Supplementary Figure 3 — Forest plot for admission to ICU. [file Image_3.TIF]

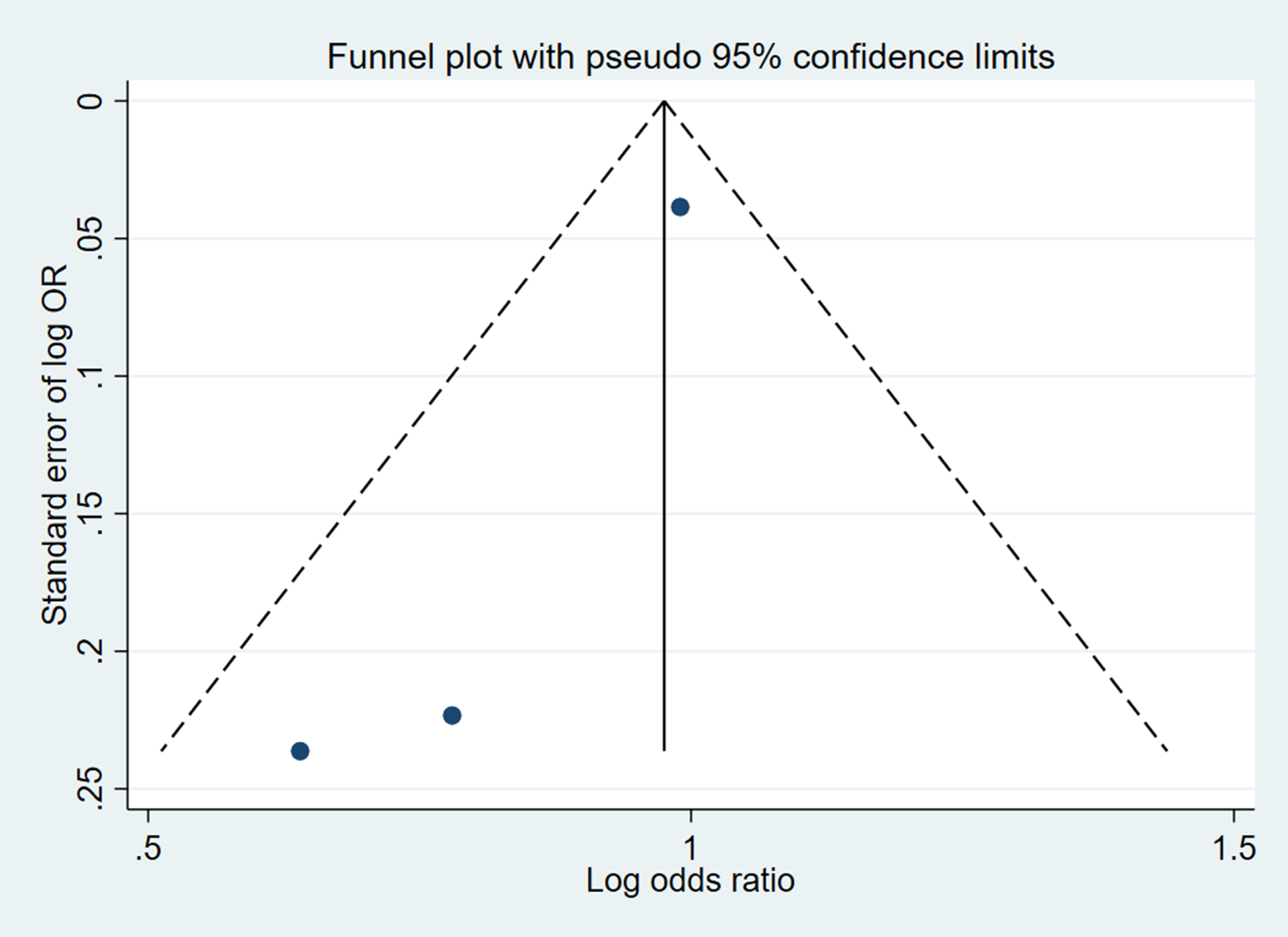

Supplement: Supplementary Figure 4 — Forest plot for readmission as an outcome. [file Image_4.TIF]
